# Methyl jasmonate mediates melatonin-induced cold tolerance of grafted watermelon plants

**DOI:** 10.1038/s41438-021-00496-0

**Published:** 2021-03-10

**Authors:** Hao Li, Yanliang Guo, Zhixiang Lan, Kai Xu, Jingjing Chang, Golam Jalal Ahammed, Jianxiang Ma, Chunhua Wei, Xian Zhang

**Affiliations:** 1 State Key Laboratory of Crop Stress Biology for Arid Areas, College of Horticulture, Northwest A&F University, 712100 Yangling, Shaanxi China; 2 College of Horticulture and Plant Protection, Henan University of Science and Technology, 471023 Luoyang, Henan China; 3 State Key Laboratory of Vegetable Germplasm Innovation, 300384 Tianjin, China

**Keywords:** Abiotic, Jasmonic acid, Plant signalling

## Abstract

Root–shoot communication has a critical role in plant adaptation to environmental stress. Grafting is widely applied to enhance the abiotic stress tolerance of many horticultural crop species; however, the signal transduction mechanism involved in this tolerance remains unknown. Here, we show that pumpkin- or figleaf gourd rootstock-enhanced cold tolerance of watermelon shoots is accompanied by increases in the accumulation of melatonin, methyl jasmonate (MeJA), and hydrogen peroxide (H_2_O_2_). Increased melatonin levels in leaves were associated with both increased melatonin in rootstocks and MeJA-induced melatonin biosynthesis in leaves of plants under cold stress. Exogenous melatonin increased the accumulation of MeJA and H_2_O_2_ and enhanced cold tolerance, while inhibition of melatonin accumulation attenuated rootstock-induced MeJA and H_2_O_2_ accumulation and cold tolerance. MeJA application induced H_2_O_2_ accumulation and cold tolerance, but inhibition of JA biosynthesis abolished rootstock- or melatonin-induced H_2_O_2_ accumulation and cold tolerance. Additionally, inhibition of H_2_O_2_ production attenuated MeJA-induced tolerance to cold stress. Taken together, our results suggest that melatonin is involved in grafting-induced cold tolerance by inducing the accumulation of MeJA and H_2_O_2_. MeJA subsequently increases melatonin accumulation, forming a self-amplifying feedback loop that leads to increased H_2_O_2_ accumulation and cold tolerance. This study reveals a novel regulatory mechanism of rootstock-induced cold tolerance.

## Introduction

As sessile organisms, plants frequently face challenges from various environmental factors throughout their life cycle. In particular, cold stress is one of the most destructive abiotic stresses due to its adverse effects on plant growth and development and subsequent negative impacts on crop productivity^1^. To adapt and survive cold exposure, plants have evolved sophisticated defense mechanisms. When a plant senses that temperature decreases via molecular sensors, the production of secondary messengers is triggered, and a set of transcriptional regulators are subsequently activated to regulate plant tolerance^2^.

At the whole-organism level, root-to-shoot communication is crucial for the increased survival of plants under environmental stress. Under drought conditions, roots produce more abscisic acid (ABA), which is then transported to the leaves to reduce water loss via stomatal closure^3^. In many horticultural crop species, grafting is widely applied to enhance plant tolerance to various environmental stresses, such as soil-borne pathogens, salt, and low temperature. Grafting-induced plant tolerance is associated with the inherent resistance of rootstocks and some rootstock-sourced signals that are transported to shoots and subsequently regulate shoot responses^4^. Therefore, grafting is also a useful tool to reveal the signaling mechanisms related to root–shoot communication. For instance, by using cucumber scions grafted onto heat-tolerant luffa rootstock, Li et al.^5^ revealed that root-produced ABA as a long-distance signal could alter the expression of heat shock protein (HSP) 70 and subsequently improve heat tolerance of the shoots.

Melatonin (*N*-acetyl-5-methoxytryptamine) was initially identified as an essential animal hormone that has regulatory roles in various biological processes^6^. In 1995, melatonin was identified in vascular plants for the first time^7,8^. A number of subsequent studies have shown phytomelatonin to be an essential regulator in plant growth and development; postharvest physiology; and defense against various environmental stresses, such as pathogen infection, drought, salinity, nutrient deficiency, and heavy metals^9–11^. In particular, the recent identification of the first phytomelatonin receptor (CAND2/PMTR1) in *Arabidopsis* has opened the door to consider melatonin a new phytohormone^12,13^. Increasing numbers of studies have indicated that melatonin enhances the cold tolerance of various plant species, including melon, tomato, watermelon, and *Arabidopsis*^14^. Moreover, some evidence has shown melatonin to be a novel long-distance signal that can be transported from roots to shoots^15–17^. Therefore, it would be interesting to investigate the involvement of melatonin in grafting-induced cold tolerance.

In plants, jasmonates (JAs) such as jasmonic acid (JA) and its methyl ester (methyl jasmonate, MeJA) act as important phytohormones that regulate multiple plant processes, such as seed germination, root growth, flowering, leaf senescence, and defense responses to various biotic and abiotic stresses^18,19^. Accumulating amounts of data have shown that JAs have a positive role in inducing plant tolerance to cold stress^19^. Cold exposure rapidly induces JA biosynthesis-related genes and subsequent JA accumulation^20^. Moreover, exogenous application of MeJA enhances *Arabidopsis* tolerance to cold, while mutants defective in JA biosynthesis or signaling exhibit hypersensitivity to cold stress^19^. Recent studies involving grafting experiments have shown that JA is involved in root and shoot communication to fine-tune plant responses to shoot wounding and root-knot nematodes^21,22^. However, little is known about the involvement of JA in root–shoot communication under cold stress.

Watermelon is a widely cultivated vegetable crop species worldwide and is very sensitive to cold stress^23^. Grafting onto pumpkin can induce watermelon tolerance to cold^24,25^. Our recent study suggests that melatonin application to the roots can confer cold tolerance to the shoot via xylem transport, and such induction involves the expression of several genes involved in JA signaling and the production of H_2_O_2_^17^, an important secondary messenger in the cold response^26^. This raises the possibility that melatonin has an important role in rootstock-induced shoot tolerance against the cold by interacting with JA and H_2_O_2_. To test this assumption, the present study evaluated the roles of melatonin, MeJA, and H_2_O_2_ and their interaction in rootstock-induced cold tolerance. Our results provide convincing evidence that melatonin-induced cold tolerance of grafted watermelon plants essentially involves MeJA and H_2_O_2_ signaling. This study provides novel insight into the mechanism of rootstock-induced cold tolerance in cucurbits.

## Materials and methods

### Plant materials

Three cucurbit species, namely, watermelon (*Citrullus lanatus* (Thunb.) Matsum. & Nakai cv. Nongkeda No. 5, *Cl*), pumpkin (*Cucurbita moschata* Duch cv. Weizhen No. 1, *Cm*), and figleaf gourd (*Cucurbita ficifolia* Bouché, *Cf*), were used in the current study. Germinated seeds of *Cl*, *Cm*, and *Cf* as rootstocks were sown into plastic pots (7 × 7 × 7.5 cm (length × width × height, respectively)) filled with 210 cm^3^ of commercial peat-based compost, and 7 d later, germinated seeds of *Cl* (as scions) were sown. Top insertion grafts were performed when the cotyledons of watermelon (as scions) had fully expanded^27^. The resulting three groups of grafted seedlings were designated *Cl/Cl*, *Cl/Cm*, and *Cl/Cf*; *Cl/Cl* plants were used as control. The plants were grown in growth chambers with a temperature of 25/18 °C (day/night), a 12-h photoperiod, and photosynthetic photon flux density (PPFD) of 400 μmol m^−2^ s^−1^. The seedlings were watered every 2 d and supplied with Hoagland’s nutrient solution every 3 d.

### Experimental design

*Experiment 1*. To evaluate the effects of different rootstocks on scion tolerance to cold stress, *Cl*/*Cl, Cl*/*Cm*, and *Cl*/*Cf* plants with four true leaves were transferred into growth chambers maintained at 25 °C for the control treatment or 4 °C for cold treatment. At 0, 6, 12, 18, and 24 h after cold treatment, the relative expression levels of *C-REPEAT BINDING FACTOR 1* (*ClCBF1*) and *ClCBF2* were measured. At 36 h after cold treatment, the maximum photochemical efficiency of PSII (*Fv/Fm*) and the relative electrical conductivity (REC) were measured. At 12 h after cold treatment, root or leaf samples were harvested for biochemical assays.

*Experiment 2*. To determine the effects of exogenous melatonin, MeJA, and H_2_O_2_ on the cold tolerance of *Cl*/*Cl* seedlings, the leaves were first sprayed with distilled water (as a control), melatonin at 150 μM^17^, MeJA at 200 μM^19^, or H_2_O_2_ at 2 mM^5^. Melatonin (Sigma-Aldrich, St. Louis, MO, USA) or MeJA (Sigma-Aldrich) was dissolved in ethanol followed by dilution with distilled water at a ratio of 1/10,000 (v/v). Each plant was sprayed with 20 mL of the respective chemical solution or distilled water (as a control). At 12 h after pretreatment with melatonin, MeJA, or H_2_O_2_, the plants were subjected to 4 °C temperature. To block the synthesis or accumulation of melatonin, JA, or H_2_O_2_ in *Cl*/*Cl* plants, the leaves were pretreated with 100 μM *p*-chlorophenylalanine (CPA, a melatonin synthesis inhibitor)^28,29^, 5 mM diethyldithiocarbamic acid (DIECA, a JA biosynthesis inhibitor)^30^, or 20 μM diphenylene iodonium (DPI, an inhibitor of NADPH oxidase, which produces H_2_O_2_)^31^. Eight hours later, the plants were sprayed with melatonin or MeJA, and 12 h later, they were exposed to 4 °C.

*Experiment 3*. To assess the involvement of melatonin, MeJA, and H_2_O_2_ and their interactions in rootstock-induced cold tolerance, the leaves of *Cl*/*Cm* and *Cl*/*Cf* seedlings were sprayed with CPA, DIECA, or DPI 8 h prior to cold exposure. The leaves were sampled at 12 h, whereas the *Fv/Fm* and REC were measured at 36 h after cold exposure.

### Cold tolerance assays

The *Fv/Fm* was measured using an imaging pulse-amplitude-modulated (PAM) chlorophyll fluorometer (Heinz Walz, GmbH, Effeltrich, Germany) according to the method described by Li et al.^5^. The REC was determined and calculated as described by Hong et al.^32^.

### Melatonin measurements

Melatonin was extracted and measured as described previously^33^. Frozen samples (0.5 g) were homogenized in 5 mL of acetone–methanol buffer (acetone/methanol/water = 89/10/1) on ice. After centrifugation (5,000 g, 4 °C) for 10 min, 0.5 mL of trichloric acid (1%) was added to the supernatant for protein precipitation. After centrifugation at 10,000×*g* for 10 min at 4 °C, the supernatants were used to quantify melatonin levels using an ELISA kit (Shanghai Lanpai Biotechnology Co., Ltd., Shanghai, China) according to the manufacturer’s instructions.

### Quantification of MeJA

MeJA was extracted as described by Pan et al.^34^. For the extraction of MeJA, 0.5 g of frozen leaf samples was homogenized in 5 mL of 1-propanol/H_2_O/concentrated HCl (2/1/0.002, v/v/v) and subsequently incubated overnight. The extracts and 5 mL of dichloromethane were then mixed together, after which the mixture was shaken at 4 °C for 30 min. After centrifugation, the obtained lower phase was dried under a stream of N_2_ gas. The residue was then dissolved in methanol. MeJA concentrations were analyzed using an ELISA kit (China Agricultural University, Beijing, China) following the manufacturer’s instructions.

### Analysis of H_2_O_2_

H_2_O_2_ in the leaf samples was measured as described by Bellincampi et al.^35^. Briefly, the leaf material (0.3 g) was homogenized in 2 mL of 0.2 M HClO_4_. After centrifugation (10,000×*g*, 4 °C) for 15 min, an aliquot of supernatant (0.5 mL) was added to 0.5 mL of assay reagent consisting of 500 μM ammonium ferrous sulfate, 200 μM xylenol orange, 50 mM H_2_SO_4_, and 200 mM sorbitol. After incubation for 1 h, the absorbance at OD_560_ was recorded. DAB staining of H_2_O_2_ was performed following the protocol described by Thordal-Christensen et al.^36^.

### qRT-PCR analysis

Total RNA was extracted from the leaves using an RNA extraction kit (AxGen, Union City, CA, USA). Residual DNA was removed using a DNase Mini Kit (Qiagen, Hilden, Germany). The isolated total RNA (1 μg) was reverse transcribed using a ReverTra Ace qPCR RT Kit (Toyobo, Osaka, Japan). qRT-PCR was then performed using an iCycler iQ Multicolor PCR Detection System (Bio-Rad, Hercules, CA, USA) as described by Li et al.^17^. The primers used for qRT-PCR are listed in Supplementary Table S1. The relative expression levels were standardized to those of watermelon *β-ACTIN* and were calculated as described by Livak and Schmittgen^37,38^.

### Statistical analysis

The experiment was carried out in accordance with a completely randomized design, with three independent biological replicates. Each replicate included 15 plants. For statistical analysis, the data were analyzed using variance (ANOVA), and *P* values < 0.05 were considered statistically significant according to Tukey’s test.

## Results

### Pumpkin and figleaf gourd rootstocks induced cold tolerance in watermelon shoots

**Fig. 1:**
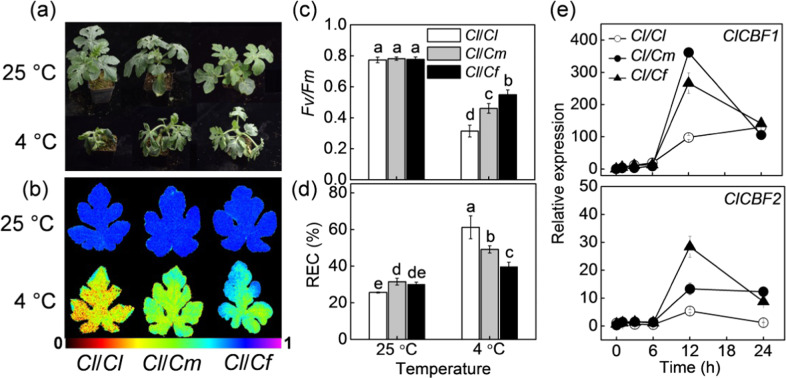
Pumpkin or figleaf gourd rootstocks induced cold tolerance and the *C-REPEAT BINDING FACTOR* (*CBF*) transcripts in watermelon shoots. Watermelon plants grafted with roots of watermelon (*Cl*/*Cl*), pumpkin (*Cl*/*Cm*), and figleaf gourd (*Cl*/*Cf*) were treated with cold at 4 °C for 36 h. **a** Chilling phenotypes. **b** Images of the maximum photochemical efficiency of PSII (*Fv/Fm*). The false-color code depicted at the bottom of the image ranges from 0 (black) to 1 (purple). **c** The average values of *Fv/Fm*. **d** Relative electrical conductivity (REC). The data from **a** to **d** were measured at 36 h after cold treatment. **e** Changes in the relative expression of *ClCBF1* and *ClCBF2* at 0, 6, 12, 18, and 24 h, respectively, after cold treatment. The data are the means of three replicates (±SDs). The means denoted with different letters differed significantly at *P* < 0.05

We first compared the leaf phenotypes, *Fv/Fm* values, and REC of watermelon plants grafted onto the rootstocks of watermelon (*Cl*/*Cl*), pumpkin (*Cl*/*Cm*), and figleaf gourd (*Cl*/*Cf*). Under normal conditions at 25 °C, no significant differences in plant growth or *Fv/Fm* were observed among the different grafted plants, although the REC of the *Cl/Cm* and *Cl/Cf* plants was slightly higher than that of the *Cl/Cl* plants (Fig. 1a–d). Cold stress caused severe leaf wilting, a significant decrease in *Fv/Fm,* and an increase in REC of the *Cl/Cl* plants. However, pumpkin and especially figleaf gourd as rootstocks alleviated the cold-induced wilting, *Fv/Fm* decrease, and REC increase in watermelon leaves. For example, the *Fv/Fm* of *Cl/Cm* and *Cl/Cf* plants were 0.46 and 0.55, respectively, which was much higher than that (0.31) of *Cl/Cl* plants at 36 h after cold exposure.

The relative expression of *ClCBF1* and *ClCBF2* was upregulated after cold treatment, and the highest peak occurred at 12 h in nearly all of the grafted plants; however, the highest expression of *ClCBF1* in the *Cl/Cl* plants occurred at 24 h after cold exposure (Fig. 1e). Importantly, *Cl/Cm* and *Cl/Cf* plants showed more significant increases in transcript levels of *ClCBF1* and *ClCBF2* than did *Cl/Cl* plants after cold exposure. For example, *ClCBF1* transcript levels were upregulated 98.1-, 361.6-, and 266.4-fold in *Cl/Cl*, *Cl/Cm*, and *Cl/Cf* plants, respectively, while the *ClCBF2* transcripts were upregulated 5.3-, 13.3-, and 28.4-fold, respectively, at 12 h after cold stress.

### Different rootstocks induced melatonin accumulation differently in leaves of watermelon plants under cold stress

**Fig. 2:**
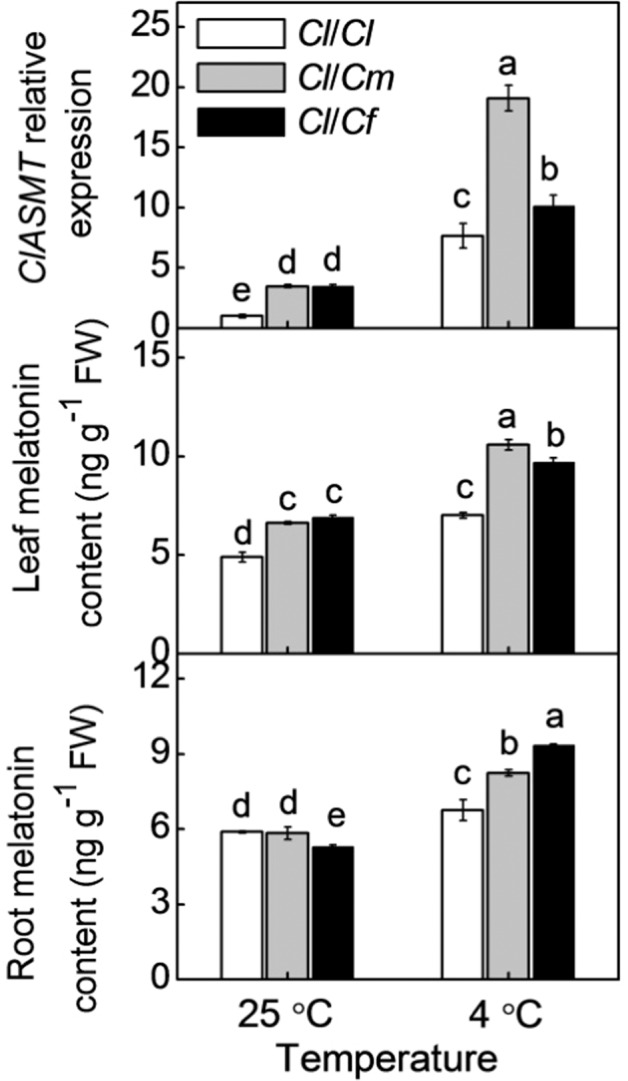
The transcript abundance of *N-ACETYLSEROTONIN O-METHYLTRANSFERASE* (*ClASMT*) and melatonin accumulation in response to different rootstocks and cold stress. The seedlings were treated as described in Fig. 1. Root and leaf samples were harvested at 12 h after the cold exposure. The data are presented as the means of three replicates (±SDs). The means denoted with different letters differ significantly at *P* < 0.05

At the optimum growth temperature, the melatonin contents in pumpkin and figleaf gourd roots were similar to and lower than those in watermelon roots, respectively (Fig. 2). However, both pumpkin and figleaf gourd as rootstocks increased the melatonin contents and increased the relative expression of *N-ACETYLSEROTONIN O-METHYLTRANSFERASE* (*ClASMT*), a key gene involved in melatonin biosynthesis, in the leaves. Cold stress-induced melatonin accumulation in both the roots and leaves and upregulated the relative expression of *ClASMT* in the leaves of all grafted seedlings at 12 h after cold treatment. Interestingly, the melatonin contents in the roots of *Cl/Cm* and especially *Cl/Cf* were obviously higher than those in the *Cl/Cl* roots after cold exposure. Moreover, the levels of melatonin and *ClASMT* transcripts in the *Cl/Cm* and *Cl/Cf* leaves were also higher than those in the *Cl/Cl* leaves.

### Pumpkin and figleaf gourd rootstocks induced MeJA and H_2_O_2_ accumulation in leaves of watermelon plants under cold stress

**Fig. 3:**
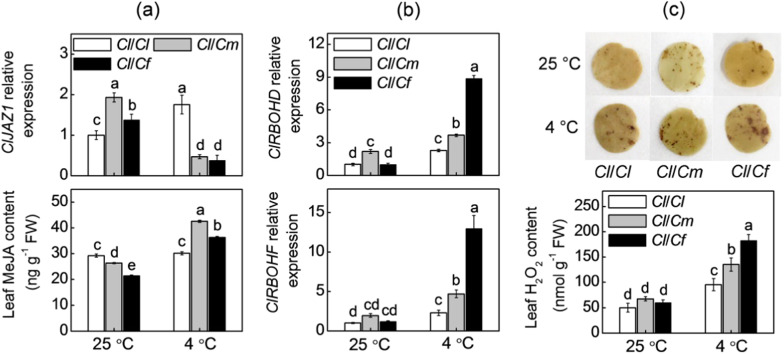
Accumulation of methyl jasmonate (MeJA) and H_2_O_2_ in leaves in response to different rootstocks and cold stress. The treatments were the same as those described in Fig. 1. Leaf samples were harvested at 12 h after the cold exposure. **a** Transcript abundances of *JASMONATE ZIM-DOMAIN* (*JAZ*) *1* and MeJA contents. **b** Transcript abundances of *RESPIRATORY BURST OXIDASE HOMOLOG* (*RBOH*) *D* and *RBOHF*. **c** DAB staining and H_2_O_2_ contents. The data are presented as the means of three replicates (±SDs). The means denoted with different letters differ significantly at *P* < 0.05

Under normal conditions at 25 °C, pumpkin or figleaf gourd as rootstocks decreased the MeJA contents but increased the relative expression of *JASMONATE ZIM-DOMAIN 1* (*ClJAZ1*, a repressor gene involving in JA signaling) in watermelon leaves (Fig. 3a). The MeJA content in *Cl/Cl* leaves was almost unchanged after exposure to cold stress; however, that in *Cl/Cm* and *Cl/Cf* leaves significantly increased. The MeJA contents in the *Cl/Cm* and *Cl/Cf* leaves were 40.4% and 19.7% higher than those in the *Cl/Cl* leaves, respectively, at 12 h after cold stress. The relative expression of *ClJAZ1* was upregulated in the *Cl/Cl* leaves but downregulated in the *Cl/Cm* and *Cl/Cf* leaves of plants under cold stress. Unexpectedly, no significant differences were observed in root MeJA content among the different grafted plants under cold stress (Supplementary Fig. S1).

The H_2_O_2_ generated by *RESPIRATORY BURST OXIDASE HOMOLOG* (*RBOH*) functions as an important signaling molecule in regulating plant tolerance to cold stress. Thus, we investigated whether H_2_O_2_ is involved in rootstock-induced cold tolerance. At optimum growth temperatures, pumpkin as a rootstock induced the accumulation of *ClRBOHD* transcripts in watermelon leaves compared to watermelon rootstocks (Fig. 3b). However, there were no significant differences in *ClRBOHF* transcripts or H_2_O_2_ content among any of the grafted plants (Fig. 3b, c). Exposure to 4 °C increased H_2_O_2_ accumulation and *ClRBOHD* and *ClRBOHF* transcript levels in all grafted plants. The leaves of *Cl*/*Cm* and especially *Cl*/*Cf* plants showed higher H_2_O_2_ levels and *ClRBOHD/F* transcripts than did those of *Cl*/*Cm* under cold stress. For example, the H_2_O_2_ contents in the *Cl/Cm* and *Cl/Cf* leaves were 41.8% and 91.3% higher than those in the *Cl/Cl* leaves, respectively, after cold exposure.

### Role of melatonin in rootstock-induced accumulation of MeJA and H_2_O_2_ and cold tolerance

**Fig. 4:**
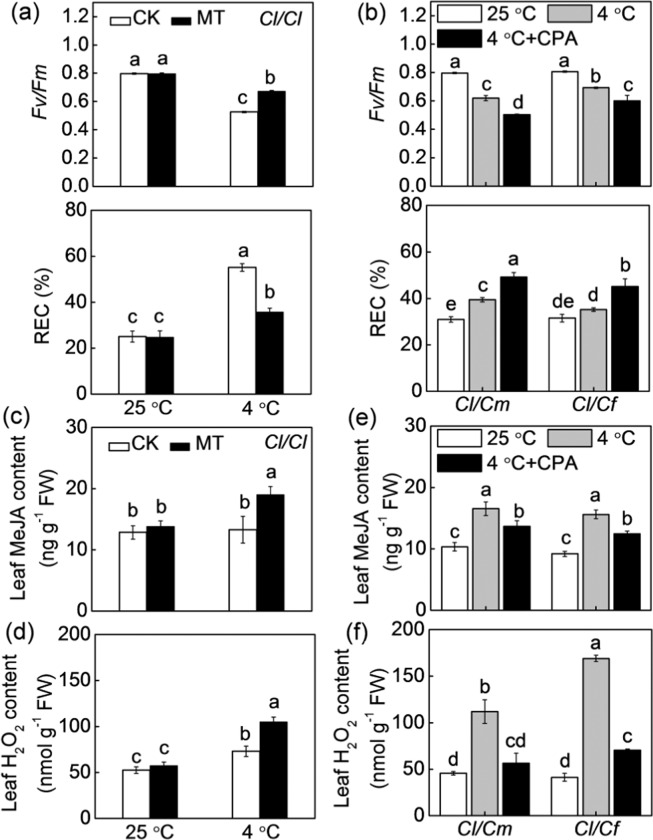
Involvement of melatonin in pumpkin or figleaf gourd rootstock-induced cold tolerance and accumulation of MeJA and H_2_O_2_ in watermelon leaves. **a** Changes in the *Fv/Fm* and REC of watermelon plants grafted onto watermelon (*Cl*/*Cl*). **b** Changes in the *Fv/Fm* and REC of watermelon plants grafted onto pumpkin (*Cl*/*Cm*) or figleaf gourd (*Cl*/*Cf*). **c** MeJA and **d** H_2_O_2_ contents in *Cl*/*Cl* plants. **e** MeJA and **f** H_2_O_2_ contents in *Cl*/*Cm* and *Cl*/*Cf* plants. For **a**, **c**, and **d**, the leaves were sprayed with melatonin (150 μM) 12 h prior to cold exposure at 4 °C. For **b**, **e**, and **f**, the leaves were sprayed with *p*-chlorophenylalanine (CPA, 100 μM) 8 h prior to cold exposure at 4 °C. The data from **a** and **b** were determined at 36 h after cold exposure. MeJA and H_2_O_2_ were measured at 12 h after cold treatment. The data are the means of three replicates (±SDs). The means denoted with different letters differ significantly at *P* < 0.05

To evaluate the role of melatonin in rootstock-induced cold tolerance, we first analyzed the effect of exogenous melatonin on the cold tolerance of *Cl*/*Cl* plants. Melatonin (150 μM) pretreatment alleviated the cold-induced *Fv/Fm* decrease and REC increase (Fig. 4a). In melatonin-pretreated plants, the *Fv/Fm* was 14.8% higher while the REC was 35.0% lower after exposure to cold stress than in the control plants. As CPA application can prevent melatonin biosynthesis^29^, we thus evaluated the effect of CPA on the cold tolerance of *Cl/Cm* and *Cl/Cf* plants. Pretreatment with CPA attenuated the pumpkin- and figleaf gourd rootstock-induced alleviation of cold stress, as reflected by the *Fv/Fm* decrease and REC increase (Fig. 4b).

At the optimum growth temperatures, exogenous melatonin application did not induce significant changes in MeJA and H_2_O_2_ levels in *Cl*/*Cl* leaves (Fig. 4c, d). However, the contents of MeJA and H_2_O_2_ increased by 42.9% and 44.1%, respectively, in response to melatonin under cold stress. More importantly, pretreatment with CPA attenuated or completely blocked pumpkin- and figleaf gourd rootstock-induced increases in MeJA and H_2_O_2_ accumulation in the leaves (Fig. 4e, f). These data suggest that melatonin has an important role in pumpkin- or figleaf gourd rootstock-induced accumulation of MeJA and H_2_O_2_ and cold tolerance.

### Roles of MeJA and H_2_O_2_ in melatonin- or rootstock-induced cold tolerance

**Fig. 5:**
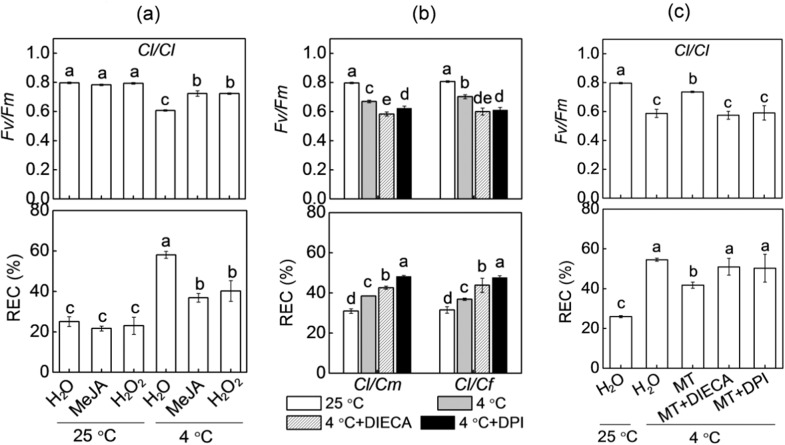
Involvement of MeJA and H_2_O_2_ in melatonin- or pumpkin or figleaf gourd rootstock-induced cold tolerance in watermelon leaves. **a** Changes in the *Fv/Fm* and REC of watermelon plants grafted onto watermelon (*Cl*/*Cl*). Plant leaves were sprayed with MeJA (200 μM) or H_2_O_2_ (2 mM) 12 h prior to cold exposure at 4 °C. **b** Changes in the *Fv/Fm* and REC in watermelon plants grafted onto pumpkin (*Cl*/*Cm*) or figleaf gourd (*Cl*/*Cf*). Plant leaves were sprayed with diethyldithiocarbamic acid (DIECA, 5 mM) or diphenylene iodonium (DPI, 20 μM) 8 h prior to cold exposure at 4 °C. **c** Changes in the *Fv/Fm* and REC of *Cl*/*Cl* plants. The plant leaves were sprayed with DIECA or DPI 8 h prior to melatonin treatment. Twelve hours later, the plants were exposed to cold at 4 °C. The data are presented as the means of three replicates (±SDs). The means denoted with different letters differ significantly at *P* < 0.05

To evaluate the roles of MeJA and H_2_O_2_ in cold tolerance acquired by grafting and melatonin, we first evaluated the effects of foliar applications of MeJA or H_2_O_2_ on the cold tolerance of *Cl*/*Cl* plants. Both MeJA (200 μM) and H_2_O_2_ (2 mM) application alleviated the cold-induced decrease in *Fv/Fm* and increase in REC of *Cl*/*Cl* plants (Fig. 5a). Pretreatment with DIECA (an inhibitor of JA biosynthesis, 5 mM) or DPI (an inhibitor of H_2_O_2_ production, 20 μM) attenuated or abolished the pumpkin- or figleaf gourd rootstock-induced alleviation of cold stress, as reflected by a decline in *Fv/Fm* and an increase in REC (Fig. 5b). Furthermore, DIECA or DPI application also prevented the melatonin-induced *Fv/Fm* increase and REC decrease after exposure to cold stress (Fig. 5c). Taken together, these results suggest that MeJA and H_2_O_2_ are involved in melatonin- or rootstock-induced cold tolerance.

### Role of MeJA in rootstock-induced accumulation of melatonin and H_2_O_2_

**Fig. 6:**
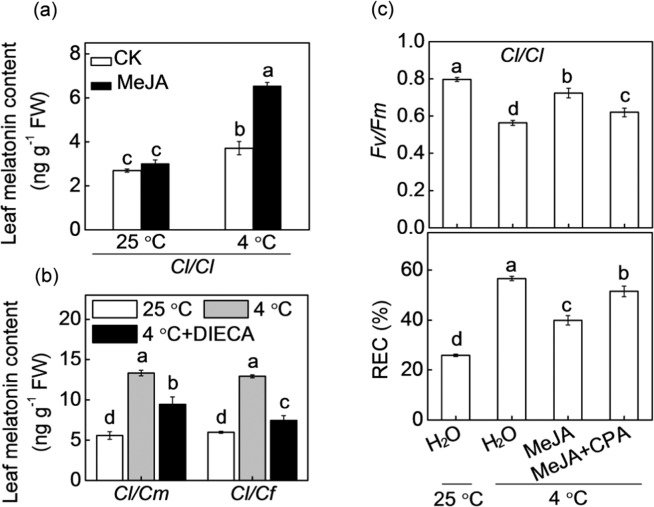
Involvement of melatonin in MeJA-induced cold tolerance of grafted watermelon plants. **a** Changes in melatonin contents in watermelon plants grafted onto watermelon rootstock (*Cl*/*Cl*). Seedlings were pretreated with MeJA (200 μM) and then subjected to 4 °C for 12 h. **b** Changes in melatonin contents in watermelon plants grafted onto pumpkin (*Cl*/*Cm*) or figleaf gourd (*Cl*/*Cf*). Leaves were pretreated with DIECA 8 h prior to cold exposure at 4 °C for 12 h. **c** Changes in the *Fv/Fm* and REC of *Cl*/*Cl* plants. Plants were pretreated with CPA, and 8 h later, the plants were sprayed with MeJA. Twelve hours later, the plants were subjected to 4 °C for 36 h. The data are presented as the means of three replicates (±SDs). The means denoted with different letters differ significantly at *P* < 0.05

To evaluate whether MeJA regulates melatonin biosynthesis in a feedback manner, we analyzed the response of melatonin to MeJA. The application of MeJA significantly increased melatonin accumulations in the *Cl*/*Cl* leaves after cold stress (Fig. 6a), while DIECA attenuated the pumpkin- or figleaf gourd rootstock-induced increase in melatonin contents (Fig. 6b). Moreover, CPA pretreatment attenuated the MeJA-induced *Fv/Fm* increase and REC decrease of *Cl*/*Cl* leaves of plants under cold stress (Fig. 6c).

**Fig. 7:**
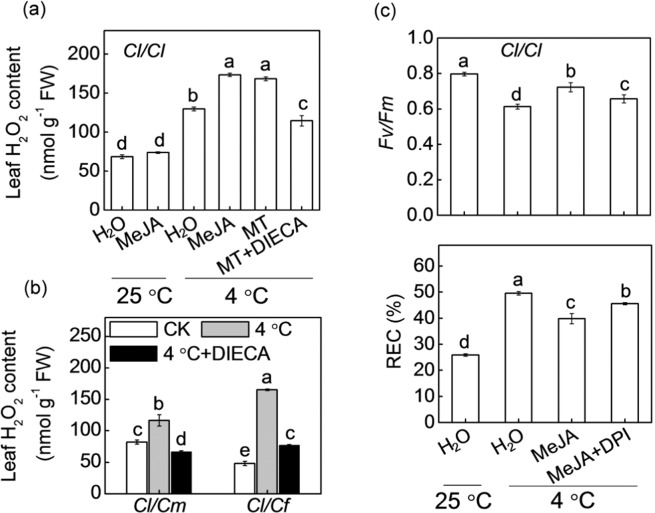
Involvement of H_2_O_2_ in MeJA-induced cold tolerance of grafted watermelon plants. **a** Changes in H_2_O_2_ contents in watermelon plants grafted onto watermelon (*Cl*/*Cl*). Seedlings were pretreated with or without DIECA, and 8 h later, the plants were sprayed with MeJA or melatonin. After 12 h, the plants were subjected to 4 °C for 12 h. **b** Changes in H_2_O_2_ contents in watermelon plants grafted onto pumpkin (*Cl*/*Cm*) or figleaf gourd (*Cl*/*Cf*). Plant leaves were sprayed with DIECA, and 8 h later, the plants were subjected to 4 °C for 12 h. **c** Changes in *Fv/Fm* and REC of *Cl*/*Cl* plants. Plants were pretreated with DPI, and 8 h later, the plants were sprayed with MeJA. After 12 h, the plants were subjected to 4 °C for 36 h. The data are presented as the means of three replicates (±SDs). The means denoted with different letters differ significantly at *P* < 0.05

To further characterize the role of H_2_O_2_ in MeJA-enhanced cold tolerance, we first evaluated the effects of MeJA on H_2_O_2_ accumulation in *Cl*/*Cl* plants. MeJA application induced an increase in H_2_O_2_ accumulation under cold stress (Fig. 7a), while DIECA pretreatment completely abolished the melatonin-induced increase in H_2_O_2_. Similarly, DIECA also completely blocked the pumpkin- and figleaf gourd rootstock-induced increase in H_2_O_2_ accumulation under cold stress (Fig. 7b). Furthermore, DPI pretreatment attenuated the MeJA-induced alleviation of cold stress, as reflected by the *Fv/Fm* decrease and REC increase (Fig. 7c).

## Discussion

### Melatonin is involved in rootstock-induced cold tolerance

Grafting onto tolerant rootstocks is well known to enhance plant tolerance to various environmental stresses, such as soil-borne pathogens, cold, and salinity. Consistent with the findings of previous studies^24,25^, we found that the rootstocks of pumpkin or figleaf gourd enhanced watermelon tolerance to cold stress (Fig. 1). By using RNA-seq analysis, Xu et al.^39^ found that pumpkin rootstocks could alter the expression of *COLD-RESPONSIVE* (*COR*) genes in shoots of watermelon plants under chilling stress. CBFs, the major activators of a subset of *COR* genes, have essential roles in modulating plant responses to cold stress^40^. In the present study, pumpkin or figleaf gourd as rootstocks significantly increased the transcripts of *ClCBF1* and *ClCBF2* in watermelon leaves after cold exposure (Fig. 1e). Therefore, it is plausible that some long-distance signal(s) originating from rootstocks are involved in inducing the shoot response to cold stress.

Increasing amounts of evidence have demonstrated that melatonin can induce cold tolerance in various plant species, and such induction is associated with the upregulated expression of *CBF*s^41,42^. Here, we observed that pumpkin or figleaf gourd rootstocks increased the accumulation of melatonin in leaves of watermelon plants under cold stress (Fig. 2). Furthermore, exogenous melatonin improved the cold tolerance of *Cl*/*Cl* plants, while inhibition of melatonin biosynthesis by CPA attenuated pumpkin- or figleaf gourd rootstock-induced cold tolerance (Fig. 4a, b). These results suggest that melatonin has an important role in pumpkin- or figleaf gourd rootstock-induced cold tolerance. It is worth noting that the increased melatonin contents in *Cl*/*Cm* or *Cl*/*Cm* leaves were accompanied by greater increases in melatonin contents in pumpkin or figleaf gourd rootstocks, respectively, after exposure to cold stress. Our previous study provided evidence that, as a long-distance signal, melatonin can be transported from roots to shoots via the xylem, subsequently inducing shoot tolerance to cold stress^17^. These data thus suggest that under cold stress, pumpkin- or figleaf gourd rootstock-sourced melatonin may act as a long-distance signal that induces melatonin accumulation and cold tolerance in watermelon leaves.

### Melatonin and MeJA function together in a self-amplifying feedback loop in rootstock-induced cold tolerance

Like melatonin, JAs have important roles in regulating plant tolerance to cold stress^43^. Under cold stress, JAs trigger the degradation of JAZ proteins, which releases INDUCER OF CBF EXPRESSION (ICE) from repression and then activates CBF-mediated transcriptional regulatory cascades^20^. In the current study, we observed that pumpkin and figleaf gourd rootstocks led to increased MeJA accumulation but decreased *ClJAZ1* transcript levels in leaves of watermelon plants under cold stress (Fig. 3a). Moreover, exogenous MeJA improved the cold tolerance of *Cl*/*Cl* plants (Fig. 5a), while inhibition of JA biosynthesis by DIECA decreased the cold tolerance of *Cl*/*Cm* and *Cl*/*Cf* plants (Fig. 5b), suggesting that MeJA is involved in rootstock-induced cold tolerance.

Recently, crosstalk between melatonin and MeJA in plant defense against biotic stress has been revealed in some studies. For instance, Liu et al.^44^ revealed that melatonin induces tomato fruit resistance to *Botrytis cinerea* by activating the JA signaling pathway. However, the crosstalk between melatonin and MeJA in abiotic stress responses remains largely unknown. Our results showed that melatonin and MeJA increased the accumulation of each other in the leaves of *Cl*/*Cl* plants under cold stress (Figs. 4c and 6a), while pretreatment with CPA or DIECA attenuated the pumpkin- or figleaf gourd rootstock-induced increase in MeJA or melatonin, respectively (Figs. 4e and 6b). Furthermore, melatonin- and MeJA-induced cold tolerance was attenuated or completely blocked by DIECA and CPA, respectively, in *Cl*/*Cl* plants (Figs. 5c and 6c). Taken together, these results suggest that melatonin and MeJA function together in a self-amplifying feedback loop, in which melatonin induces MeJA accumulation and MeJA subsequently increases melatonin accumulation during the cold response of *Cl*/*Cm* and *Cl*/*Cf* plants. Such crosstalk between melatonin and MeJA during the cold response is similar to that between ABA and H_2_O_2_ in plant responses to heat and oxidative stresses^45^.

### MeJA mediates melatonin- and rootstock-induced H_2_O_2_ accumulation and subsequent cold tolerance

As an important secondary messenger, the H_2_O_2_ generated by *RBOH* has an essential role in regulating plant tolerance against various abiotic stresses, including cold stress^26,46^. In our study, pumpkin or figleaf gourd as rootstocks increased the accumulation of H_2_O_2_, as well as the levels of *ClRBOHD* and *ClRBOHF* transcripts under cold stress (Fig. 3b, c). Additional experiments showed that exogenous application of H_2_O_2_ enhanced the cold tolerance of *Cl*/*Cl* plants, while inhibition of H_2_O_2_ generation by DPI prevented pumpkin- or figleaf gourd rootstock-induced cold tolerance (Fig. 5a, b). These results indicate that H_2_O_2_ is involved in rootstock-induced cold tolerance, and these findings are consistent with previous findings that H_2_O_2_ is involved in rootstock-induced heat tolerance of cucumber plants^5^.

Melatonin is a well-documented antioxidant that can effectively remove reactive oxygen species (ROS) and consequently alleviate oxidative stress^47^. However, accumulating studies have shown that melatonin can increase H_2_O_2_ accumulation in plant tissues, and as a key signaling molecule, H_2_O_2_ mediates melatonin-induced lateral root formation, stomatal closure, and tolerance to environmental stresses^12,48,49^. In agreement with these findings, our findings also showed that melatonin increased the H_2_O_2_ accumulation in *Cl*/*Cl* leaves, while pretreatment with CPA completely abolished the pumpkin- or figleaf gourd rootstock-induced increase in H_2_O_2_ under cold stress (Fig. 4d, f). Moreover, DPI pretreatment completely blocked the melatonin-induced cold tolerance of *Cl*/*Cl* plants (Fig. 5c). Thus, it is apparent that H_2_O_2_ mediates melatonin-induced cold tolerance of *Cl*/*Cm* and *Cl*/*Cf* plants.

It has been well established that JA and H_2_O_2_ function synergistically to regulate multiple physiological processes, such as leaf senescence, drought tolerance, stomatal closure, and wound-induced responses, and JA functions upstream of H_2_O_2_^50,51^. Consistent with the data of these studies, our data show that MeJA mediates the melatonin-induced cold tolerance of *Cl*/*Cm* and *Cl*/*Cf* plants by inducing H_2_O_2_ accumulation. This conclusion is based on the following evidence: (1) foliar application of MeJA increased H_2_O_2_ accumulation in *Cl*/*Cl* plants in response to cold (Fig. 7a), while H_2_O_2_ application failed to induce an increase in MeJA accumulation (Supplementary Fig. S2); (2) pretreatment with DIECA prevented melatonin- or rootstock-induced H_2_O_2_ accumulation (Fig. 7a, b); and (3) application of DPI attenuated the MeJA-induced cold tolerance of *Cl*/*Cl* plants (Fig. 7c).

**Fig. 8:**
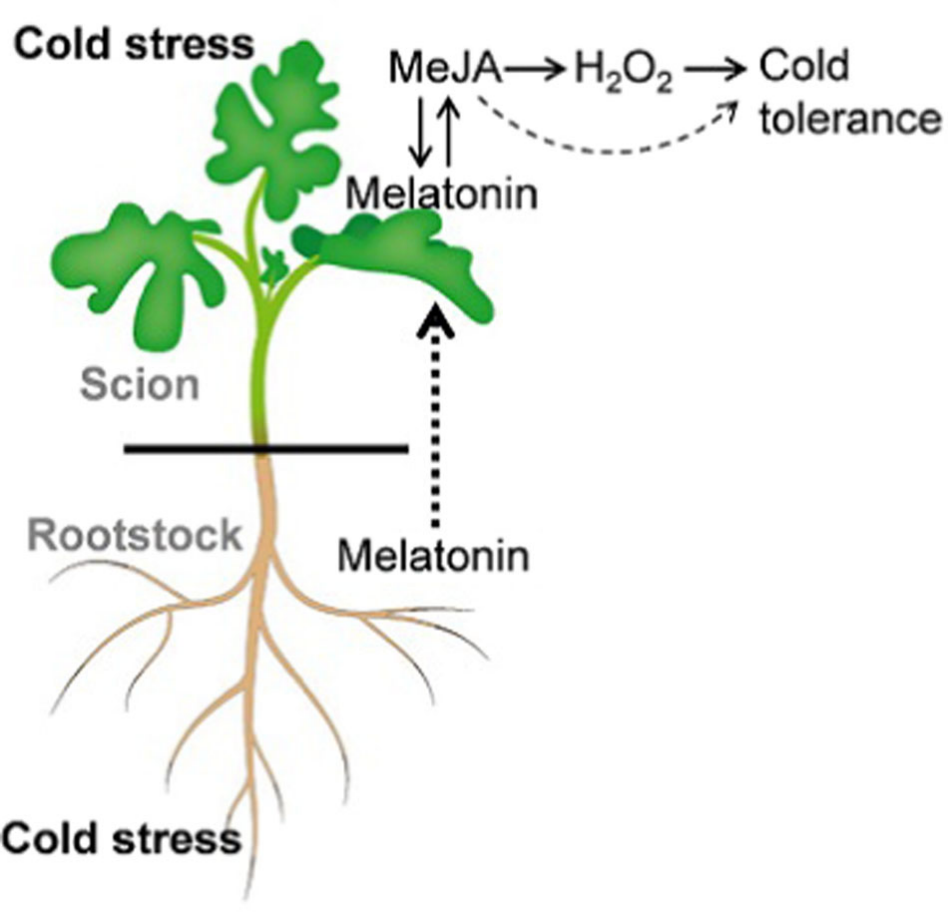
Model depicting the role of methyl jasmonate (MeJA) in melatonin-induced H_2_O_2_ accumulation and cold tolerance in watermelon plants grafted onto pumpkin or figleaf gourd rootstocks. When grafted watermelon plants suffer cold stress, rootstock-sourced melatonin may act as a long-distance signal that induces melatonin accumulation in leaves. Melatonin accumulation in leaves induces MeJA accumulation, which in turn increases melatonin accumulation, forming a self-amplifying feedback loop. MeJA further triggers H_2_O_2_ generation and subsequently enhances cold tolerance

To date, the mechanisms underlying rootstock-induced shoot tolerance to cold stress are unclear; however, we have demonstrated in the present study that, as a potential root-to-shoot signal, melatonin is involved in rootstock-induced cold tolerance. The rootstock-promoted melatonin accumulation in leaves induces MeJA accumulation, which in turn increases melatonin accumulation, forming a self-amplifying feedback loop. MeJA further triggers H_2_O_2_ generation and subsequently enhances cold tolerance (Fig. 8). Thus, melatonin-induced increases in MeJA and subsequent H_2_O_2_ have an essential role in rootstock-scion communication in response to cold stress.

## Supplementary information

Supporting information Figure S1, Figure S2, Table S1
