# Supplementary material for: Methyl jasmonate mediates melatonin-induced cold tolerance of grafted watermelon plants
Source: Hortic Res. 2021 Mar 10;8:57. doi: 10.1038/s41438-021-00496-0 (PMC7943586; doi:10.1038/s41438-021-00496-0)
Supplement: Supplementary file 1 — Supporting information Figure S1, Figure S2, Table S1 [file 41438_2021_496_MOESM1_ESM.docx]

**
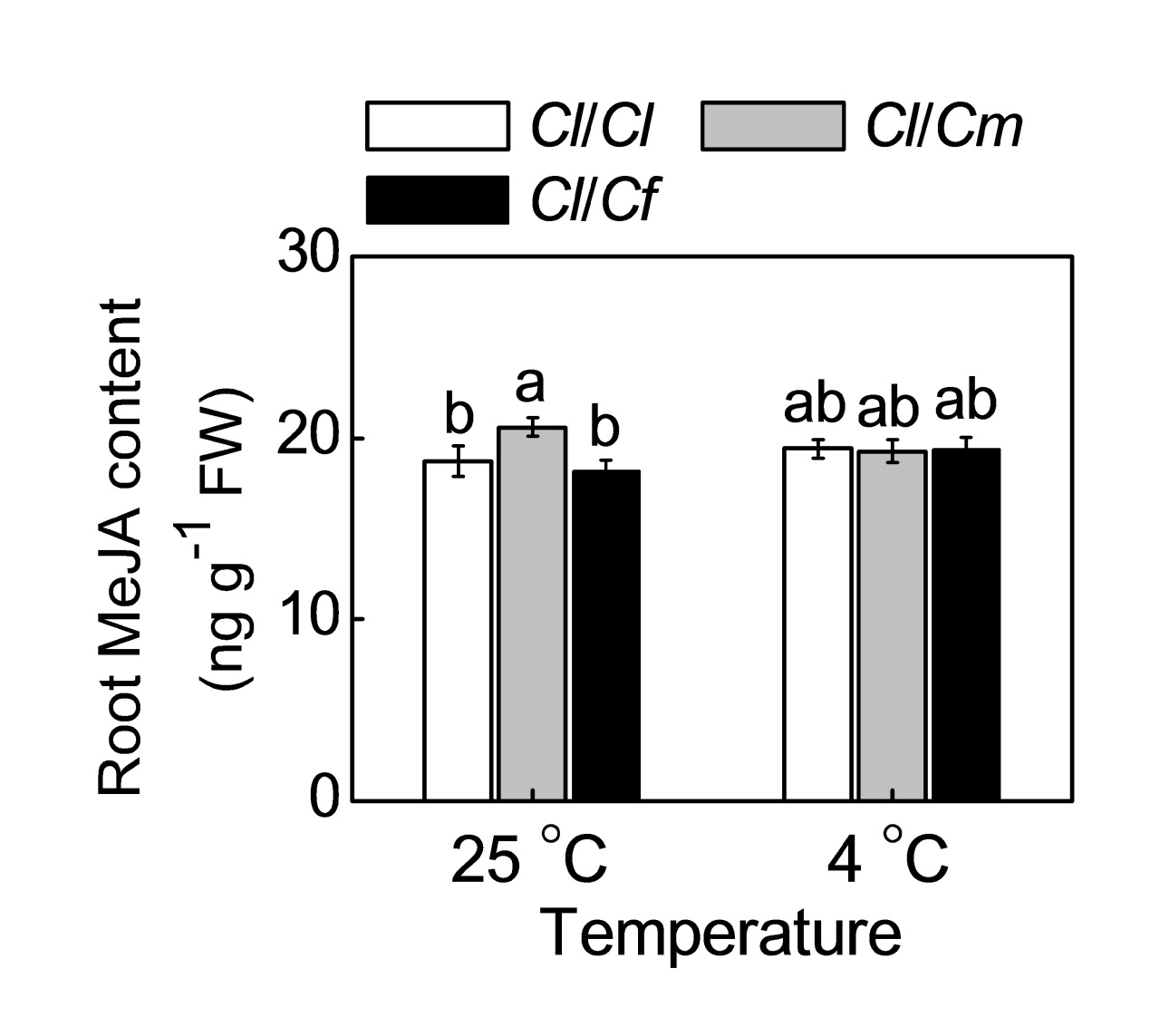
**

**Fig. S1** The response of methyl jasmonate (MeJA) accumulation to cold stress in roots of watermelon plants grafted onto watermelon (*Cl*/*Cl*), pumpkin (*Cl*/*Cm*), or figleaf gourd (*Cl*/*Cf*).

**
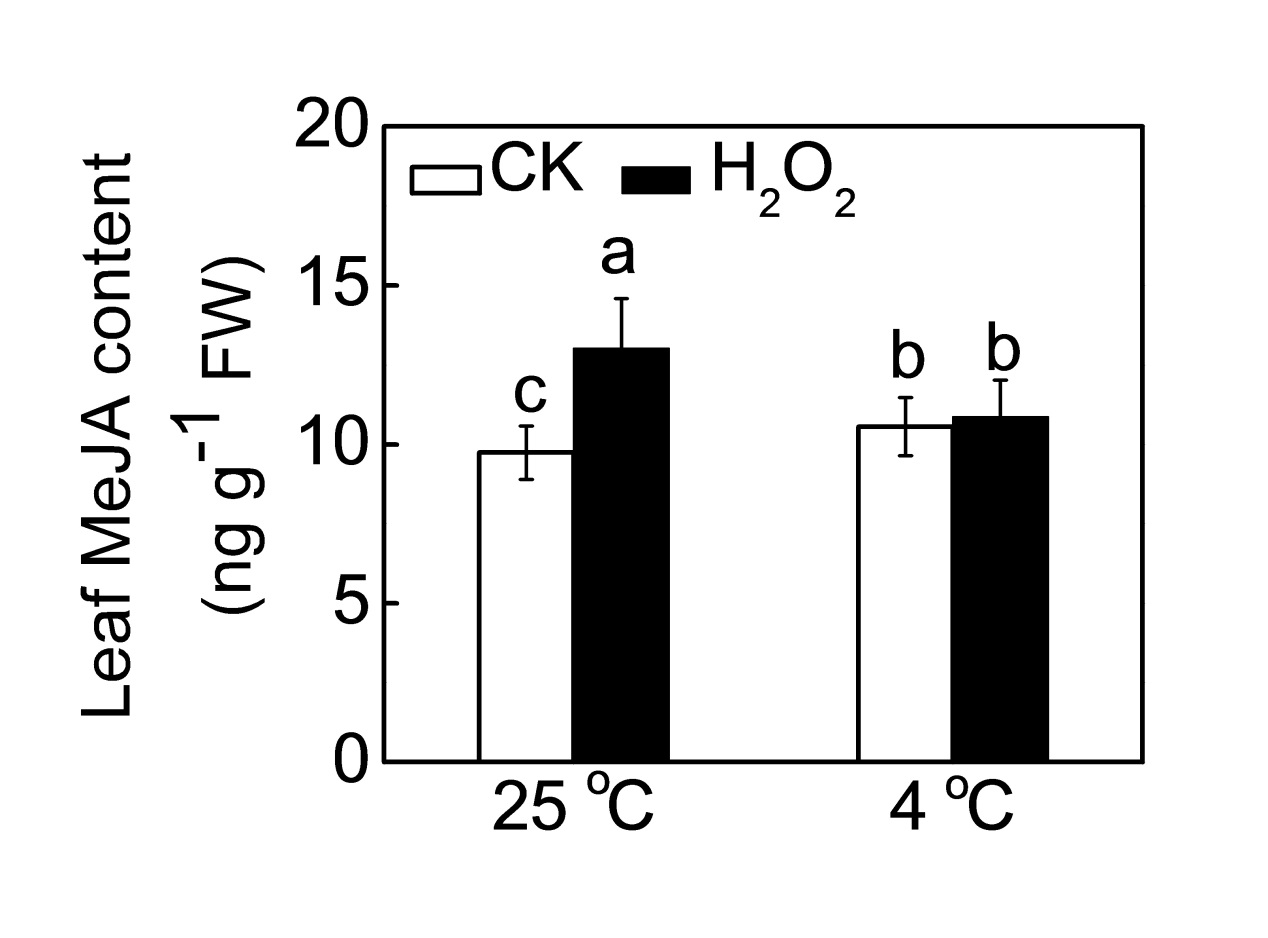
**

**Fig. S2** The effects of H_2_O_2_ on methyl jasmonate (MeJA) contents in leaves of self-grafted watermelon plants under cold stress.

**Table S1** Primers used for Real-time PCR analysis

| **Gene ID or accessions** | **Forward sequence (5’-3’)** | **Rreverse sequence (5’-3’)** | **Gene description** |
| --- | --- | --- | --- |
| Cla007792  Cla006212  Cla011488  Cla010664  Cla012536  Cla017196  Cla011409 | CCATGTATGTTGCCATCCAG  TGCTGACGACTCAAGGACGGAA  GGCGGCGAATGAGTGTAAGGAT  TCGCCACCAAGGGAGTCATTCA  TGGAACCAACAATCGCCAGAGA  ACGAGGTAGCGGAAATGGACGA  TGGACATTCTGCTGGTGCTGGA | GGATAGCATGGGGTAGAGCA  GCTGAGGTGGAGGCAACATCAT  CTGGCAAGCAACATCGGCATAT  GCACAGCAGTGGACCTTGAAAC  GCTTTCGCCAGTCGGTTTAGAA  GCATTGCTATCAACGCCGACCT  CGACGCCATTGCTCTTACTGCT | *β-actin*  *C-repeat binding factor (CBF) 1*  *CBF2*  *Acetylserotonin methyltransferase*  *Jasmonate ZIM-Domain 1*  *respiratory burst oxidase homolog* (*RBOH*) *D RBOHF* |
